# A single-aortotomy end-to-side technique simplifies murine aortic transplantation

**DOI:** 10.1371/journal.pone.0356149

**Published:** 2026-10-08

**Authors:** Hao Dun, Maura Sticco-Ivins, Gianni Bonnici, Hannah Beatty, Benjamin J. Kopecky

**Affiliations:** 1 Department of Medicine, Division of Cardiology, University of Colorado Anschutz Medical Campus, Aurora, Colorado, United States of America; 2 Department of Immunology, University of Colorado Anschutz Medical Campus, Aurora, Colorado, United States of America; Universidad Santa Paula, COSTA RICA

## Abstract

**Background:**

Transplant arteriosclerosis remains a major cause of late graft failure after solid organ transplantation. Murine aortic transplantation models have provided important insights into the mechanisms underlying chronic allograft rejection, but the surgical procedure remains technically demanding. We developed a modified end-to-side aortic transplantation technique utilizing a single recipient aortotomy to simplify vascular anastomosis in mice.

**Methods:**

Recipient infrarenal aortas were occluded proximally and distally using microvascular clamps. A single longitudinal aortotomy was created in the recipient infrarenal aorta. The proximal and distal ends of a donor thoracic aortic graft were independently anastomosed end-to-side to this single opening. Following completion of the anastomoses, a central ligature was placed between the donor graft inlet and outlet tracts to direct blood flow through the graft. Operative outcomes were compared with those of the conventional (two-aortotomy) end-to-side technique.

**Results:**

Donor harvest times were similar between groups. Recipient preparation time was shorter with the single-aortotomy technique (10.07 ± 0.95 vs. 10.87 ± 1.17 min, p = 0.033). Anastomosis time was significantly reduced compared with the conventional technique (14.73 ± 1.24 vs. 20.40 ± 1.57 min, p < 0.0001). Graft patency was 93% versus 87%, thrombosis occurred in one animal per group, and survival was comparable between techniques.

**Conclusion:**

A single-aortotomy end-to-side configuration significantly reduces anastomosis time while maintaining graft patency, survival, and the ability to reproduce transplant vasculopathy. This technique provides a simplified and reproducible approach for murine aortic transplantation.

## Introduction

The technique of abdominal heterotopic aortic transplantation in mice, as a model of chronic transplant rejection, was first described by Koulack et al. [1] in 1995. This method involves performing two distinct *end-to-end* vascular anastomoses, replacing a segment of the recipient infrarenal aorta with a 1.0 cm donor thoracic aortic graft (S1 Fig). However, its widespread application has been limited by the technical complexity of the vascular anastomoses, a challenge further exacerbated by size discrepancies between the donor thoracic aorta and the recipient abdominal aorta [2].

To overcome these technical challenges, the sleeve [3] and cuff [4] techniques were developed and have been increasingly adopted as alternative approaches. A comparative study demonstrated that both the sleeve and cuff techniques are superior to the conventional end-to-end suture method in murine aortic transplantation [5]. Nevertheless, despite these modifications, the procedures remain technically demanding and are particularly susceptible to thrombosis formation due to the small vessel diameter in mice.

Moreover, a major limitation of the end-to-end anastomosis approach is the requirement for complete separation of the recipient infrarenal aorta from the inferior vena cava (IVC). This step constitutes one of the most technically challenging aspects of the procedure and carries a high risk of vascular injury, which may lead to severe intraoperative bleeding.

Thus, the *end-to-side* technique emerged as a simplified approach for performing aortic transplantation in mice [2,6]. In this method, *two-aortotomies* are created in the recipient infrarenal aorta and subsequently anastomosed to the proximal and distal ends of the donor aortic graft (S2 Fig). The segment of the recipient infrarenal aorta between the two anastomoses is then transected [2] or ligated [6], thereby directing blood flow through the donor aortic conduit and ensuring graft perfusion.

Compared with the traditional end-to-end technique, *the end-to-side approach offers several advantages*. By clamping the recipient infrarenal aorta and IVC together, it eliminates the need for vessel separation, thereby minimizing the risk of bleeding. In addition, this technique permits implantation of a relatively longer donor aortic segment, providing more tissue for subsequent histological and molecular analyses. More importantly, it overcomes the challenge of size discrepancies between donor and recipient vessels, making the anastomosis technically less demanding. Consequently, the end-to-side model has been reported to achieve a success rate exceeding 90% [6].

However, the conventional end-to-side technique requires the creation of two aortotomies in the recipient infrarenal aorta (S2 Fig). An intact segment of aorta must be preserved between the two aortotomies, which substantially limits the available working length of the recipient infrarenal aorta, particularly when two vascular clamps are applied. To conserve space, some investigators [6], including our group, have used tie ligation instead of clamps to occlude the recipient aorta. Despite this modification, the improvement in available aortic length remains limited. Moreover, tie ligation may cause the aorta and IVC to collapse together when they are not separated, further complicating the anastomosis and increasing technical difficulty. Whether recipient reconstruction can be simplified further while preserving biological fidelity of the model remains unknown.

Herein, we describe a *modified single-aortotomy end-to-side* aortic transplantation technique that addresses several limitations of conventional approaches. Rather than creating two separate recipient aortotomies, a single-aortotomy is used to accommodate both the proximal and distal ends of the donor graft. Following completion of the anastomoses, a central ligature separates the inlet and outlet tracts and directs blood flow through the donor graft (S3 Fig). This configuration conserves recipient aortic working length, simplifies the operative field, and reduces the complexity of vascular reconstruction. We hypothesized that this modification would decrease operative time while maintaining graft patency, survival, and the capacity to support development of transplant vasculopathy.

## Materials and methods

### Animals

Male C57BL/6J and C57BL/6J-BM12 mice, aged 8–12 weeks, were purchased from The Jackson Laboratory (Bar Harbor, ME, USA). Animals were housed under standard conditions at the University of Colorado Anschutz Medical Campus Animal Facility. Food and water were provided ad libitum. All experimental procedures were conducted in accordance with institutional guidelines and were approved by the Institutional Animal Care and Use Committee (IACUC) of the University of Colorado Anschutz Medical Campus (Protocol #01386). Animals were monitored daily during the first postoperative week and at least three times weekly thereafter for body weight, activity, grooming behavior, mobility, wound healing, and signs of distress. Humane endpoints included >15% body weight loss, persistent hindlimb paralysis, severe lethargy, labored breathing, inability to obtain food or water, or other conditions judged by veterinary staff to compromise animal welfare.

### Experimental design

Fifteen syngeneic B6 → B6 aortic transplants were performed using the single-aortotomy end-to-side technique and compared with fifteen syngeneic B6 → B6 transplants performed using the conventional two-aortotomy end-to-side technique. Operative outcomes including donor harvest time, recipient preparation time, anastomosis time, graft patency, thrombosis, bleeding complications, and survival were compared between groups (S1 Table). To evaluate whether the modified technique supported development of transplant vasculopathy, four allogeneic B6 → BM12 transplants and four syngeneic B6 → B6 transplants were performed using the new technique and analyzed 100 days after transplantation. All procedures were performed by a single microsurgeon (HD).

### Anesthesia

Animals were administered subcutaneous extended-release buprenorphine (1 mg/kg), followed by intraperitoneal ketamine hydrochloride (80 mg/kg) and xylazine (8 mg/kg) dissolved in saline. The depth of anesthesia was assessed using a toe-pinch reflex test. Animals that withdrew their foot in response to the stimulus were considered inadequately anesthetized and were not subjected to the procedure until an adequate anesthetic depth was achieved. All mice were continuously monitored throughout anesthesia and placed on a temperature-controlled heating-pad to maintain body temperature during the procedure**.**

### Donor operation

Donor operation was performed as previously described [7]. Briefly, after laparotomy, the IVC was injected with 2.0 mL of cold saline containing heparin (100 U/mL). The inferior mesenteric artery was then transected to facilitate blood decompression. Subsequently, bilateral thoracotomy was performed along both sides of the thoracic spine, and the anterior chest wall was removed. The thymus was resected to expose the aortic arch and pulmonary artery. The heart was reflected to the right, and the left lung was removed to allow full visualization of the aortic arch and descending aorta. The parietal pleura overlying the descending aorta was bluntly dissected. A 6−0 silk ligature was placed around the descending aorta proximal to the diaphragmatic hiatus and secured. The descending aorta was transected distal to the ligature and gently elevated to permit careful dissection of the surrounding connective tissue using fine scissors, proceeding proximally toward the aortic arch.

For aortic arch procurement, the three major branches of the left subclavian artery, left common carotid artery, and innominate artery were individually isolated and transected from the arch. The ascending aorta was separated from the pulmonary artery by sharp dissection of the connective tissue and was then transected near its origin from the heart. The harvested donor aorta was immediately placed in ice-cold saline solution and stored until the recipient procedure was completed.

### Recipient operation

After adequately being anesthetized, the recipient mouse was placed in a supine position with the tail oriented toward the operator. A midline laparotomy was performed from the pubis to the xiphoid process, and the abdominal cavity was exposed using a micro-retractor. The intestines were gently exteriorized to the left side and wrapped in saline-moistened gauze.

The infrarenal aorta and IVC were carefully dissected free from the surrounding tissues using blunt dissection. Based on anatomic variability, one to two groups of lumbar vessels beneath the infrarenal aorta and IVC were identified and ligated *en bloc*. The infrarenal aorta and IVC were then sequestered together using two microvascular clamps placed sequentially just above the aortic bifurcation (distal clamp) (Fig 1A) and below the renal vessels (proximal clamp) (Fig 1B), respectively (S1 Video).

**Fig 1:**
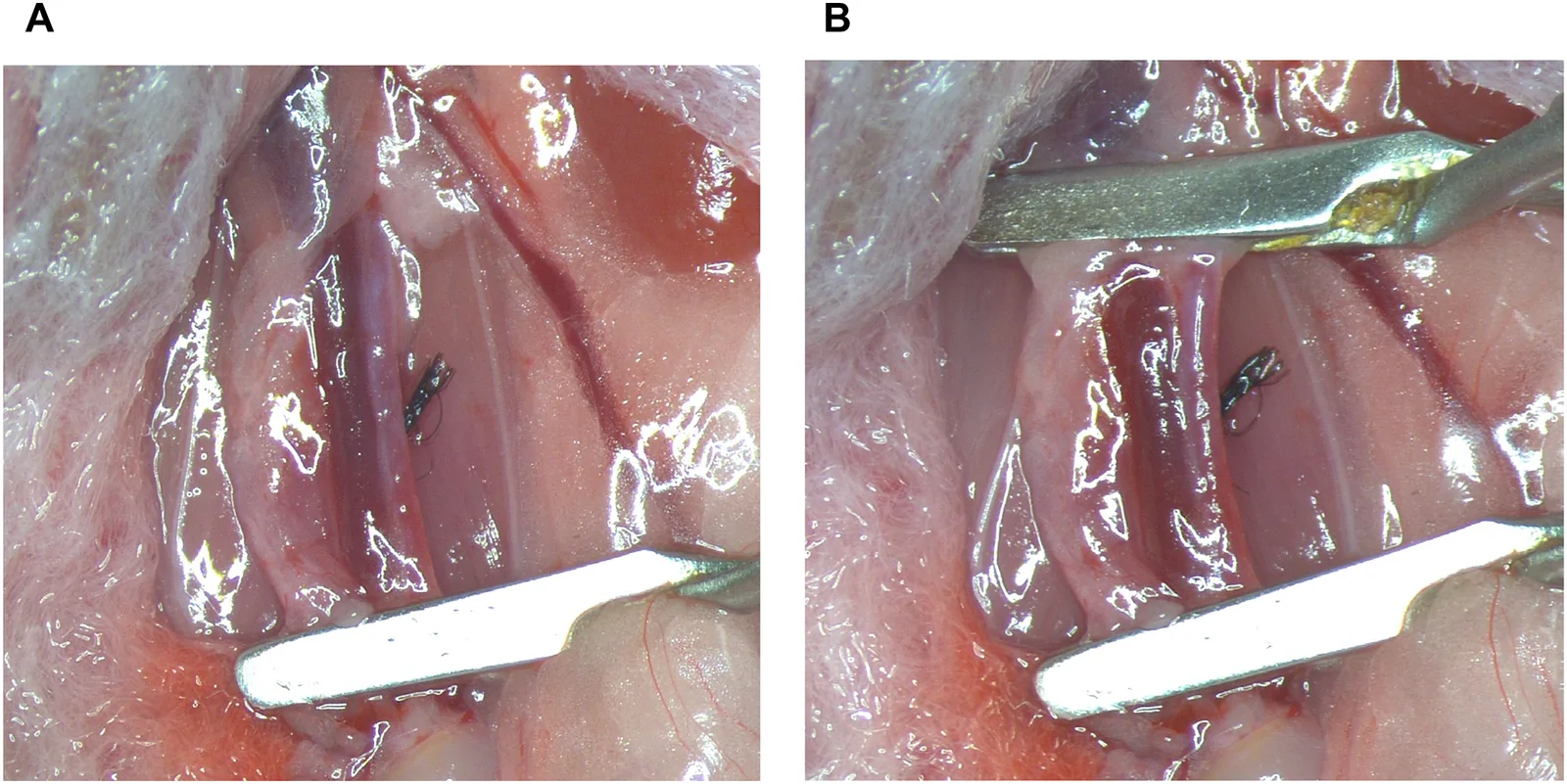
(A and B) Exposure of Aorta and IVC. The infrarenal aorta and IVC were occluded together using two microvascular clamps placed sequentially just above the aortic bifurcation (distal clamp) **(A)** and below the renal vessels (proximal clamp) **(B)**, respectively.

An aortotomy was created as previously described [7] (Fig 2A). Briefly, the tip of a 4 mm (3/8) needle was passed longitudinally through the anterior wall of the recipient aorta and partially brought out, then held in place with a needle holder. By gently elevating the needle, the segment of the anterior aortic wall overlying the needle was easily excised by cutting beneath it with fine scissors, creating an approximately 1 mm elliptical aortotomy with smooth, well-trimmed edges (S2 Video). The final aortotomy length was typically 1.0–1.5 mm depending on donor graft dimensions. The aortic lumen was flushed with saline to remove residual blood. The donor aortic graft was positioned on the left side of the recipient abdominal cavity (Fig 2B). The proximal (inlet) and distal (outlet) ends of the donor aortic graft were then anastomosed end-to-side to the recipient infrarenal aorta in a counterclockwise fashion using 10−0 nylon sutures. All anastomoses were performed using an everting approach, as both the anterior and posterior vessel walls were sutured from the external surface of the vessel.

**Fig 2:**
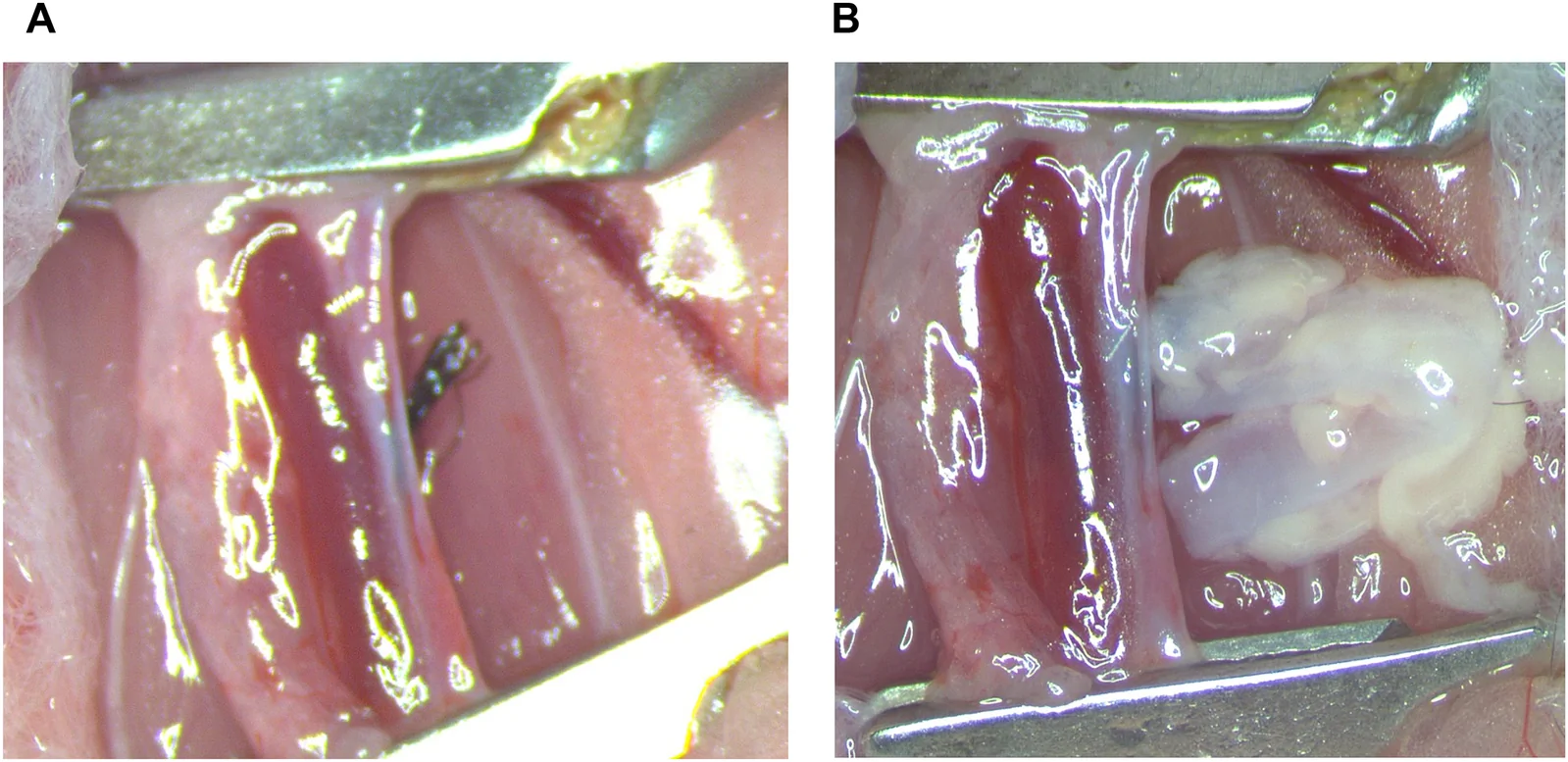
(A and B) Generation of single-aortotomy. An aortotomy was created on the sequestered infrarenal aorta **(A)**. The donor aortic graft was removed from ice-cold saline and was positioned on the left side of the recipient abdominal cavity **(B)**.

The first anchor stitch was placed at the proximal corner of the recipient aortotomy and the donor inlet tract to secure proper alignment (Fig 3A). This proximal anchor suture was retained for subsequent suturing of the right wall. The second anchor stitch was then placed at the distal corner of the recipient aortotomy and the donor outlet tract (Fig 3B). This distal anchor suture was used for suturing of the left wall following completion of the right wall anastomosis.

**Fig 3:**
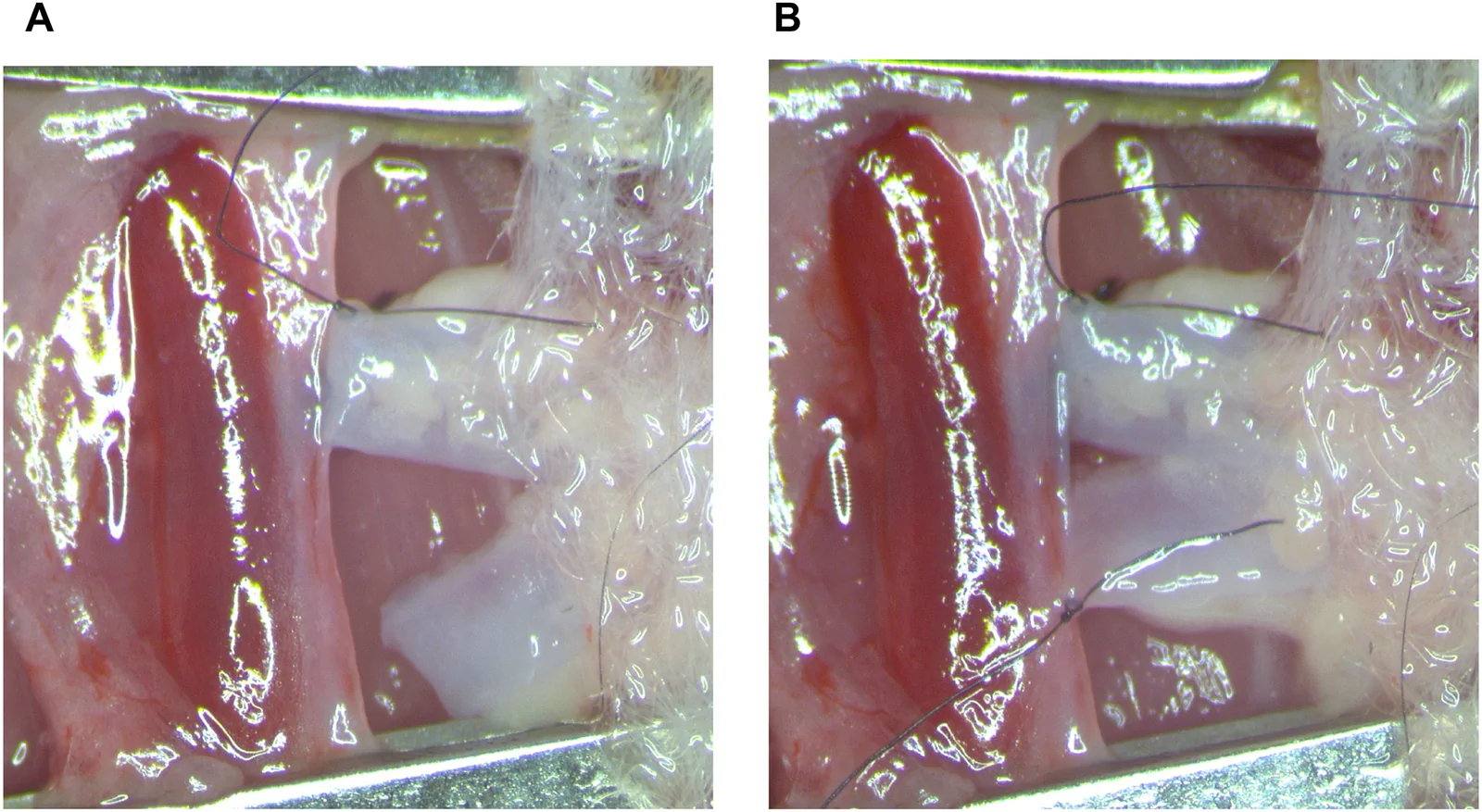
(A and B) Placement of anchor stitch. The first anchor stitch was placed at the proximal corner of the recipient aortotomy and the donor inlet tract **(A)**. The second anchor stitch was placed at the distal corner of the recipient aortotomy and the donor outlet tract to secure proper alignment **(B)**.

The proximal anchor suture was initially used to perform a running suture along the right wall outside the vessel, proceeding from proximal to distal with 3–4 stitches for both the inlet and outlet tracts of the donor aortic graft (Fig 4A). Upon reaching the distal corner, the running suture was tied to the tail of the distal anchor suture and trimmed to approximately 0.1 cm. Subsequently, the distal anchor suture was used to complete the left wall anastomosis, running from distal to proximal in a manner symmetrical to the right wall (Fig 4B).

**Fig 4:**
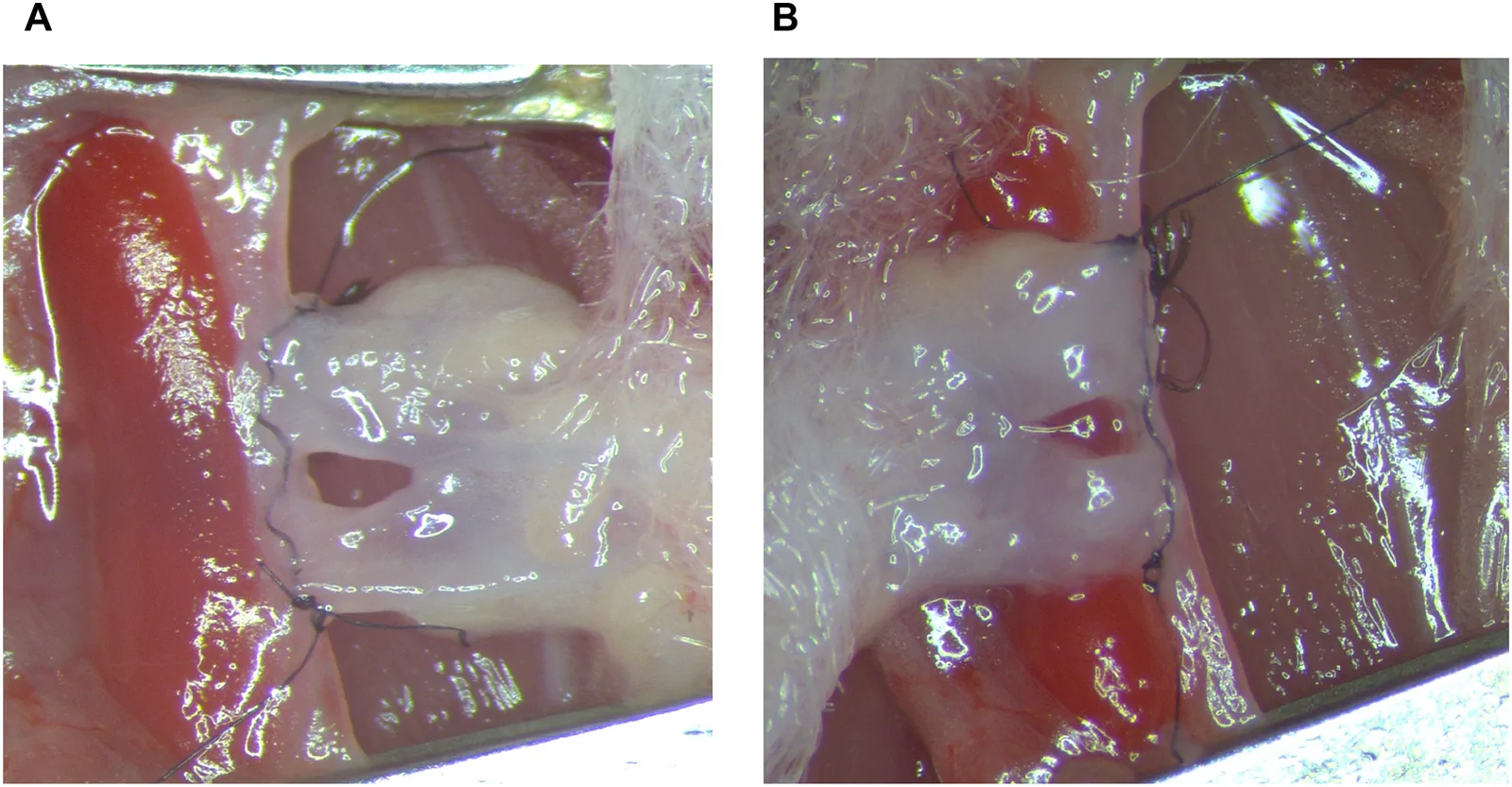
(A and B). Running sutures and anastomoses. The proximal anchor suture was initially used to perform a running suture along the right wall outside the vessel, proceeding from proximal to distal with 3-4 stitches for both the inlet and outlet tracts of the donor graft **(A)**. The distal anchor suture was then used to complete the left wall anastomosis, running from distal to proximal in a manner symmetrical to the right wall **(B)**.

After completion of the anastomoses, the recipient infrarenal aorta segment located between the inlet and outlet tracts of the donor aortic graft was circumferentially ligated with a 10−0 nylon suture (Fig 5A). This maneuver divided the anastomotic site into two isolated segments corresponding to the donor inlet and outlet tracts, thereby ensuring that blood flow passed exclusively through the aortic graft (S3 Fig).

**Fig 5:**
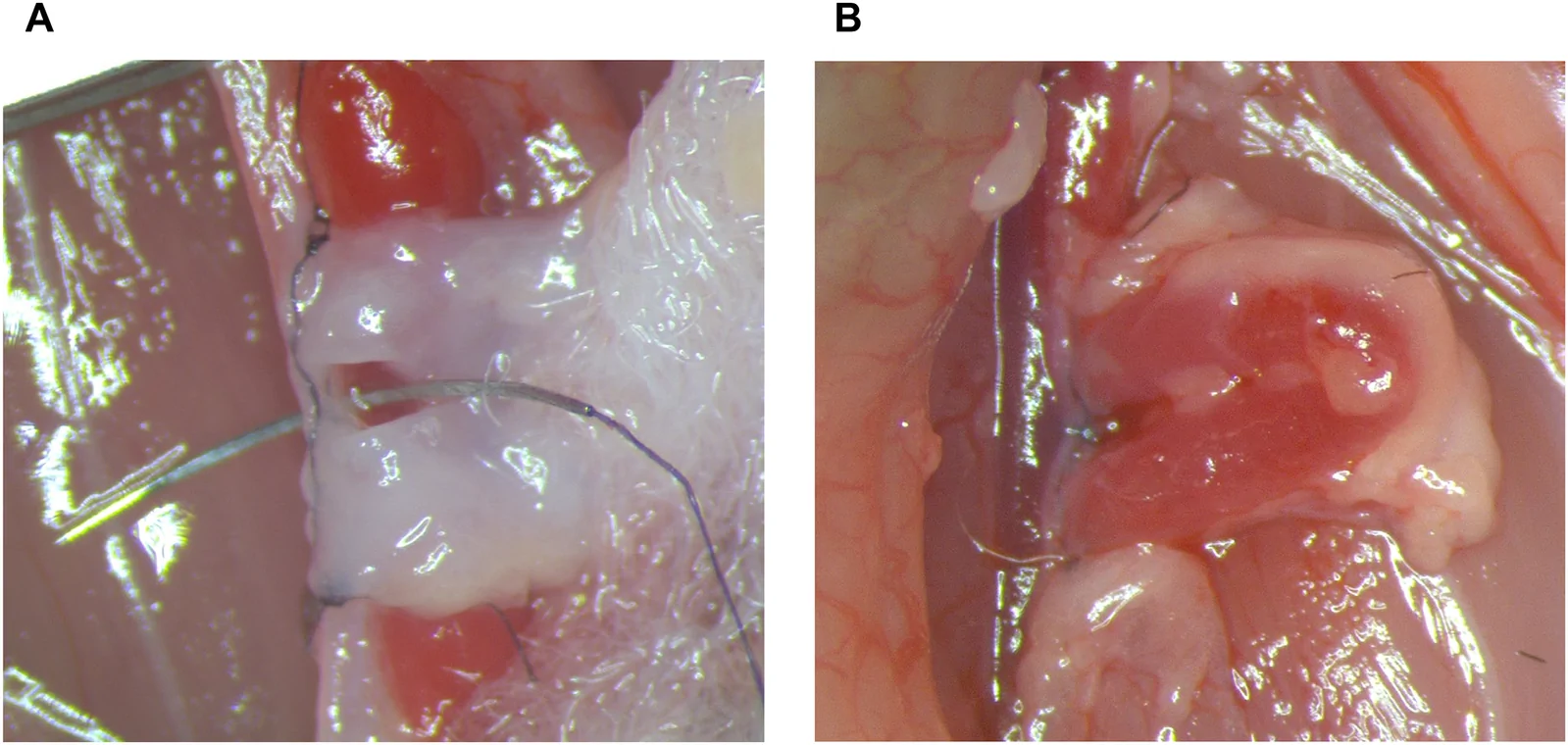
(A and B) Aorta ligation and reperfusion. The recipient infrarenal aorta was circumferentially ligated with a 10−0 nylon suture **(A)**. The distal clamp was released first, followed by the proximal clamp, and immediate restoration of pulsatile flow within the aortic graft confirmed successful reperfusion **(B)**.

Finally, the distal clamp was released first, followed by the proximal clamp. Immediate restoration of visible pulsatile flow within the donor aortic graft, along with confirmation of graft patency, indicated successful reperfusion (Fig 5B and S3 Video).

For the control group, aortic transplantation was performed using the conventional end-to-side anastomosis technique [6].

### Postoperative care

Immediately after surgery, recipient mice received 1.0 ml of sterile saline subcutaneously for fluid resuscitation. The animals were then placed in an incubator (Pet Brooder 90, Curadle ICU MXB90N) for 24 hours, maintained at 29–30°C with 50% relative humidity. Standard chow and water were provided *ad libitum*. Surgical success was defined as survival without evidence of hindlimb paralysis throughout the designated observation period. A subset of animals were followed for 100 days after transplantation, a time point previously shown to permit development of cardiac allograft vasculopathy in murine aortic transplant models [8]. Ultimately, all animals were euthanized and underwent autopsy, followed by histological analysis of the aortic grafts and relevant tissues. Euthanasia methods were approved by IACUC and included isoflurane inhalation and an approved secondary method (exsanguination). All attempts to minimize animal discomfort were taken.

### Histology

Freshly explanted donor aortas were carefully dissected to capture the anastomotic sites. Aortas were flushed with 5 mL ice cold phosphate buffered saline (PBS) and fixed overnight in 4% paraformaldehyde at 4°C. Fixed aortas were thrice rinsed with PBS and immediately processed for paraffin embedding by the histology core. For paraffin embedded tissues, 4 µm thick slices were placed on glass slides and stained for Verhoeff-Van Gieson stain (Cancer Diagnostics #SSC1135) per manufacturer’s protocol. Images were obtained on the Olympus VS200 high throughput slide scanning microscope with the 20x objective through the University of Colorado Anschutz Medical Campus Imaging Core. Images were processed and regions of interest selected on the Olyvia software. Histological evidence of vasculopathy was defined as neointimal hyperplasia associated with luminal narrowing within donor graft segments.

### Statistical analysis

All data were presented as mean with 95% confidence interval of the mean. Differences between groups were analyzed using a two-tailed Student’s *T*-test. Statistical analyses were performed using GraphPad Prism v10. A *p* value < 0.05 was considered statistically significant.

## Results

Donor aortic harvest was successfully completed in all animals. Mean donor harvest times were similar between the single-aortotomy and conventional two-aortotomy end-to-side groups (11.93 [11.40–12.47] vs. 12.13 [11.44–12.82] minutes, p = 0.627). Recipient preparation time was shorter in the single-aortotomy group (10.07 [9.58–10.56] vs. 10.87 [10.28–11.48] minutes, p = 0.033), although the absolute difference was less than one minute.

The principal procedural advantage of the modified technique was a significant reduction in donor-recipient anastomosis time. Anastomosis was completed in 14.73 [14.09–15.38] minutes using the single-aortotomy technique compared with 20.40 [19.59–21.21] minutes using the conventional two-aortotomy technique (p < 0.0001; Fig 6).

**Fig 6:**
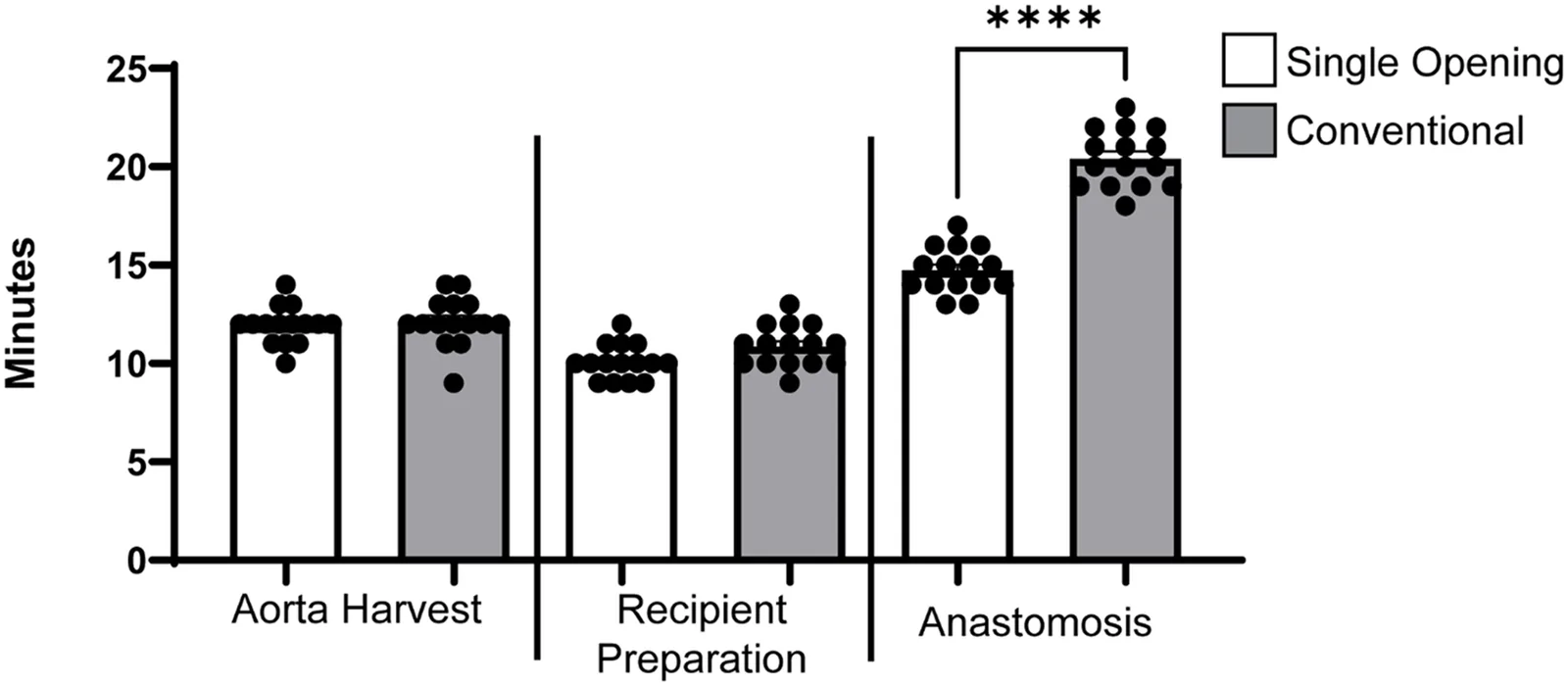
Surgical comparisons. Surgical times for aorta harvest, recipient preparation, and donor-recipient aortic anastomosis between new single-aortotomy technique compared to conventional two-aortotomy technique. P < 0.0001, n = 15 each group. Student’s t-Test.

Operative outcomes were comparable between techniques (S1 Table). Graft patency was maintained in 14 of 15 animals (93%) undergoing the single-aortotomy procedure and 13 of 15 animals (87%) undergoing the conventional two-aortotomy technique. One thrombosis occurred in each group. No bleeding complications occurred in the single-aortotomy group, whereas one bleeding complication occurred in the conventional two-aortotomy group. No animals reached humane endpoint criteria during the 100-day observation period.

To determine whether the modified single-aortotomy end-to-side technique preserves the biological utility of the model, allogeneic B6 → BM12 aortic transplants were evaluated 100 days after transplantation. Histological analysis demonstrated characteristic allograft vasculopathy with neointimal hyperplasia and luminal narrowing in donor aortic segments of allogeneic grafts (Fig 7A–7D). In contrast, syngeneic B6 → B6 grafts showed preserved vascular architecture without evidence of vasculopathy (Fig 7E–7H). Vasculopathy was confined to donor tissue and was not observed in immediately adjacent recipient aortic segments.

**Fig 7:**
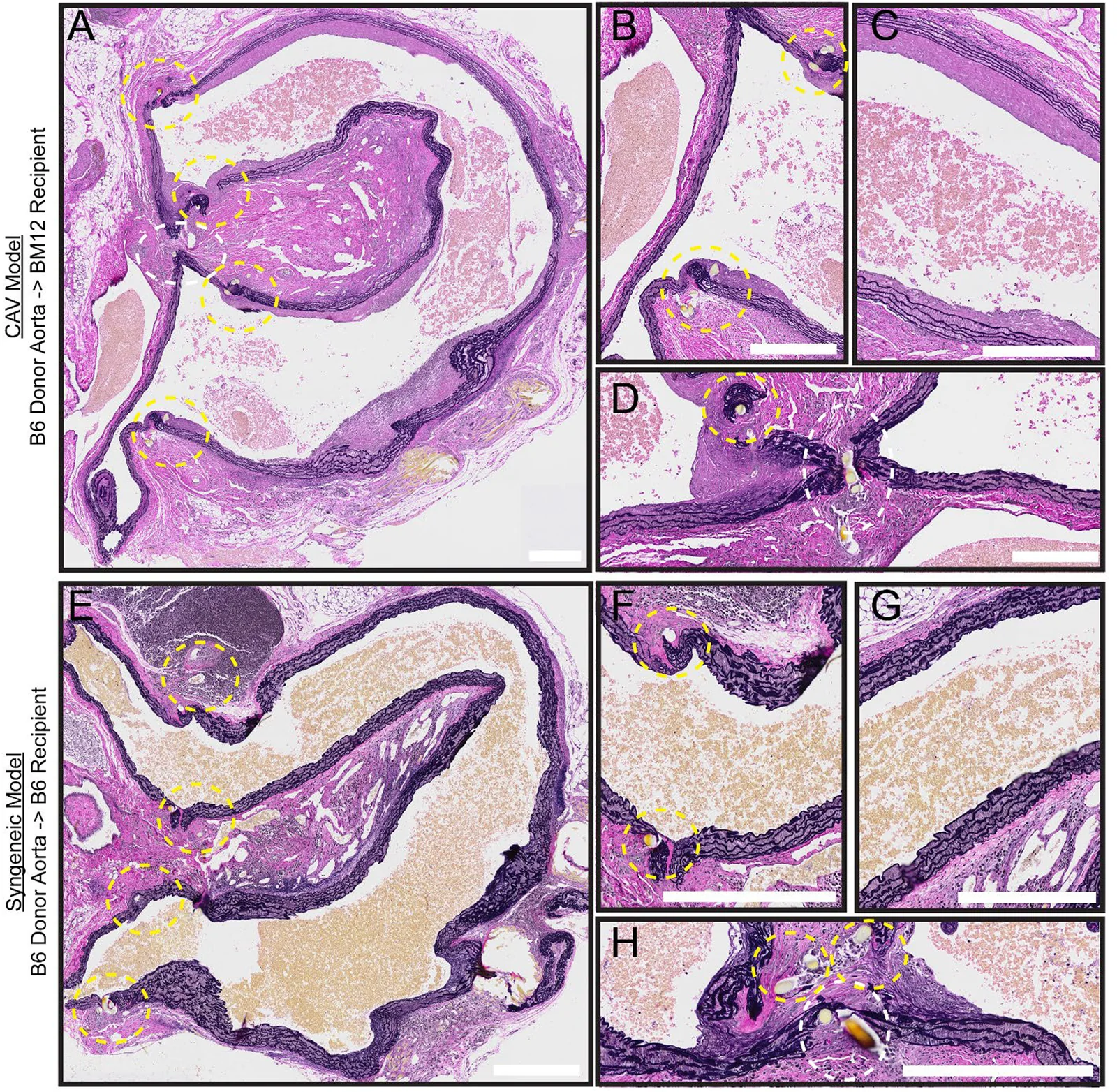
Qualitative VVG histology of CAV Model (B6 donor aorta transplanted into BM12 recipient mouse) and Syngeneic Model (B6 donor aorta transplanted into B6 recipient mouse). **(A)** Overview imaging showing proximal and **(B)** distal anastomotic sites and **(C)** development of allograft vasculopathy in the B6 portion (donor) but not BM12 (recipient) portions of the aorta. **(D)** Suture (white dashed oval) physically disrupts recipient aorta requiring all blood to divert through the allograft. **(E)** Overview of syngeneic with **(F)** proximal and distal anastomotic sites (in yellow dashed circles) and **(G)** the absence of vasculopathy in both the donor and recipient (B6) portions of aorta. **(H)** Suture (white dashed oval) physically disrupts recipient aorta requiring all blood to divert through the allograft. Scale = 500 µm.

## Discussion

The principal finding of this study is that a single-aortotomy end-to-side configuration simplifies murine aortic transplantation while preserving the essential biological characteristics of the model. Compared with the conventional two-aortotomy end-to-side technique, this approach significantly reduced anastomosis time without compromising graft patency, survival, or the development of allograft vasculopathy. These findings suggest that the single-aortotomy model represents a practical refinement of existing murine aortic transplantation techniques.

Murine aortic transplantation has evolved through successive technical modifications aimed at reducing operative complexity while maintaining experimental utility. Conventional end-to-end transplantation is limited by donor-recipient size mismatch and the need for extensive dissection to separate the aorta from the inferior vena cava, increasing the risk of bleeding. The subsequent end-to-side approach addressed many of these challenges by permitting simultaneous clamping of the aorta and IVC and avoiding complete vessel separation. However, it still requires creation of two recipient aortotomies and preservation of an intervening aortic segment, which constrains the available operative field and increases reconstructive complexity. Our modification advances this evolution by eliminating the need for two separate recipient arteriotomies and replacing them with a single shared aortotomy.

**Several technical innovations contribute to the simplification achieved by the single-aortotomy model.** The **first innovation** is the creation of a single recipient aortotomy that accommodates both the proximal (inlet) and distal (outlet) ends of the donor aortic graft. In our experience, generation of the aortotomy is most reliably performed using a 4-mm (3/8) needle passed longitudinally through the anterior wall of the recipient infrarenal aorta and secured with a needle holder. Gentle elevation of the needle creates a stable platform beneath the vessel wall, allowing the intervening tissue to be excised with fine scissors. This maneuver consistently produces a smooth elliptical opening with well-trimmed edges. Because a single opening must ultimately accommodate both donor graft ends, the initial aortotomy may occasionally require extension. When enlargement is necessary, the proximal and/or distal corners can be carefully lengthened to match the dimensions of the donor graft. However, enlargement should be performed conservatively. Excessive extension creates an oversized opening that increases the technical difficulty of the anastomosis, reduces tissue support for suturing, and may increase the risk of intraoperative bleeding.

A **second innovation** is the substantial reduction in vascular reconstruction time achieved by eliminating the need for two separate aortotomies. In the conventional end-to-side technique, two independent arteriotomies must be created, carefully positioned, and subsequently incorporated into two distinct anastomotic fields while preserving an intervening segment of recipient aorta. In contrast, the single-aortotomy configuration consolidates the reconstruction into a single operative field. This eliminates the time required to create and optimize a second recipient opening, reduces repeated vessel manipulation, simplifies graft orientation, and decreases the overall amount of vascular suturing required to complete the reconstruction. These technical advantages translated into a marked reduction in anastomosis time, with reconstruction completed approximately 28% faster than with the conventional two-aortotomy approach. Importantly, this improvement was achieved without compromising graft patency, thrombosis rates, or long-term survival, indicating that procedural simplification does not occur at the expense of surgical reliability.

A **third innovation** is preservation of recipient aortic working length. In the conventional two-aortotomy technique, an intact segment of recipient infrarenal aorta must be maintained between the proximal and distal arteriotomies. Because the available infrarenal aortic segment in mice is inherently short, this requirement significantly restricts the operative workspace, particularly when vascular clamps are present. By utilizing a single-aortotomy, no intervening recipient aortic segment must be preserved. Consequently, a greater proportion of the infrarenal aorta remains available for graft implantation and surgical manipulation. This expanded working space facilitates graft positioning, improves visualization of the operative field, and reduces technical difficulty during vascular reconstruction.

A **fourth innovation** involves the method of vascular occlusion. We apply the distal microvascular clamp first, followed by placement of the proximal clamp. This sequence allows decompression of the IVC during the clamping process. As venous blood drains from the isolated segment, the IVC progressively collapses, creating a substantially clearer operative field around the infrarenal aorta. The resulting improvement in exposure facilitates both aortotomy creation and vascular suturing. Importantly, successful decompression of the IVC requires interruption of lumbar venous inflow. One or two groups of lumbar vessels located beneath the aorta and IVC should therefore be identified and ligated before vascular clamping. Failure to interrupt these tributaries permits continued venous filling of the isolated caval segment, resulting in persistent IVC distension that complicates microsurgical manipulation. Thus, lumbar vessel ligation represents a critical technical step that contributes substantially to visualization and ease of reconstruction.

A **fifth innovation** is the adoption of a fully everting suture technique for both anterior and posterior walls of the anastomosis. In our previous murine transplantation models, posterior walls were commonly reconstructed using inverting sutures placed from within the vessel lumen, whereas anterior walls were completed using an everting approach. Although effective, this method relies on secure exposure of the posterior wall and typically requires two opposing anchor stitches for each anastomosis. In the present configuration, a single-aortotomy serves simultaneously as the anastomotic site for both donor graft ends. Because each donor segment is secured by only one anchor stitch, exposure of the posterior wall for intraluminal suturing becomes technically more challenging. We therefore adopted a fully everting approach in which both anterior and posterior walls are sutured from the external vessel surface.

Continuous suturing is performed with the needle entering through the cut edge of the donor aorta and exiting through the cut edge of the recipient aorta. This orientation provides excellent visualization of both vessel edges and facilitates precise placement of each stitch while minimizing the risk of inadvertently catching the opposite vessel wall. The anterior wall is reconstructed from the proximal to distal apex, after which the animal is rotated so that its head faces the operator, allowing the posterior wall to be completed from distal to proximal under direct visualization. This maneuver eliminates the need to flip the graft repeatedly during reconstruction and provides consistent exposure throughout the procedure. In our experience, the combination of dual anchor sutures and a fully everting continuous technique results in accurate vessel alignment, improved visualization, and efficient completion of the anastomosis within the constrained operative field created by the murine infrarenal aorta.

A critical consideration for any surgical modification is whether it preserves the biological utility of the model. Histological analysis demonstrated the expected development of allograft vasculopathy in B6 → BM12 transplants, whereas syngeneic controls maintained normal vascular architecture. Furthermore, graft patency and long-term perfusion were maintained throughout the study period. These findings indicate that the modified configuration retains the key biological features necessary for investigations of transplant arteriosclerosis and chronic allograft rejection.

Several limitations should be acknowledged. First, all procedures were performed by a single experienced microsurgeon, and the learning curve associated with the technique was not formally assessed. Second, quantitative morphometric analysis of vasculopathy was not performed. Future studies will be required to determine whether disease severity is identical between the single- and two-aortotomy configurations. Third, the hemodynamic consequences of the shared anastomotic site were not evaluated. Alterations in local flow patterns or wall shear stress could potentially influence vascular remodeling and warrant further investigation.

## Conclusion

In conclusion, the **single-aortotomy end-to-side** technique represents a meaningful surgical advance in murine aortic transplantation. By simplifying recipient reconstruction and significantly reducing anastomosis time while preserving graft function and vasculopathy development, this model provides an efficient and reproducible platform for studies of chronic allograft rejection and transplant vasculopathy.

## Supporting information

S1 Fig End-to end technique. Schematic illustration of the conventional end-to-end technique. A donor thoracic aortic graft is interposed into the recipient infrarenal aorta after resection of a corresponding aortic segment. The proximal and distal ends of the graft are anastomosed end-to-end to the recipient aorta. Following restoration of blood flow, the aortic graft becomes perfused.
(DOCX)

S2 Fig Two-aortotomy end-to-side technique. Schematic illustration of the conventional end-to-side technique. Two longitudinal aortotomies are created in the recipient infrarenal aorta. The proximal and distal ends of the donor aortic graft are independently anastomosed end-to-side to the corresponding recipient aortotomies. The intervening segment of the recipient aorta between the two anastomoses is subsequently ligated, thereby separating the proximal and distal luminal connections and establishing graft perfusion.
(DOCX)

S3 Fig Single aortotomy end-to-side technique. Schematic illustration of the novel end-to-side technique. A single longitudinal aortotomy is created in the recipient infrarenal aorta. Both the proximal and distal ends of the donor aortic graft are then anastomosed end-to-side to this single opening in a parallel configuration. The central portion of the anastomotic site is subsequently ligated, thereby separating the inflow and outflow channels and establishing graft perfusion.
(DOCX)

S1 Video (DOCX)

S2 Video (DOCX)

S3 Video Reperfusion of donor aorta. After release of clamps, blood flows into the newly anastomosed donor aorta.
(AVI)

S1 Table Comparison of the novel single-aortotomy technique and the conventional end-to-side aortic transplantation technique in mice. (DOCX)

## Abbreviations

IVC: Inferior Vena Cava
