# Supplementary material for: A single-aortotomy end-to-side technique simplifies murine aortic transplantation
Source: PLoS One. 2026 Oct 8;21(10):e0356149. doi: 10.1371/journal.pone.0356149 (PMC13649135; doi:10.1371/journal.pone.0356149)
Supplement: Supplementary file 1 [file pone.0356149.s001.docx]

**Supplementary Figure 1. End-to-End Technique. Schematic illustration of the conventional end-to-end technique.** A donor thoracic aortic graft is interposed into the recipient infrarenal aorta after resection of a corresponding aortic segment. The proximal and distal ends of the graft are anastomosed end-to-end to the recipient aorta. Following restoration of blood flow, the aortic graft becomes perfused.
