# Supplementary material for: A single-aortotomy end-to-side technique simplifies murine aortic transplantation
Source: PLoS One. 2026 Oct 8;21(10):e0356149. doi: 10.1371/journal.pone.0356149 (PMC13649135; doi:10.1371/journal.pone.0356149)
Supplement: Supplementary file 2 [file pone.0356149.s002.docx]

**Supplementary Figure 2.** **Two-Aortotomy End-to-Side Technique.** Schematic illustration of the conventional end-to-side technique. Two longitudinal aortotomies are created in the recipient infrarenal aorta. The proximal and distal ends of the donor aortic graft are independently anastomosed end-to-side to the corresponding recipient aortotomies. The intervening segment of the recipient aorta between the two anastomoses is subsequently ligated, thereby separating the proximal and distal luminal connections and establishing graft perfusion.
