# Supplementary material for: A single-aortotomy end-to-side technique simplifies murine aortic transplantation
Source: PLoS One. 2026 Oct 8;21(10):e0356149. doi: 10.1371/journal.pone.0356149 (PMC13649135; doi:10.1371/journal.pone.0356149)
Supplement: Supplementary file 3 [file pone.0356149.s003.docx]

**Supplementary Figure 3. Single-Aortotomy End-to-Side Technique. Schematic illustration of the novel end-to-side technique.** A single longitudinal aortotomy is created in the recipient infrarenal aorta. Both the proximal and distal ends of the donor aortic graft are then anastomosed end-to-side to this single opening in a parallel configuration. The central portion of the anastomotic site is subsequently ligated, thereby separating the inflow and outflow channels and establishing graft perfusion.
