# Supplementary material for: A single-aortotomy end-to-side technique simplifies murine aortic transplantation
Source: PLoS One. 2026 Oct 8;21(10):e0356149. doi: 10.1371/journal.pone.0356149 (PMC13649135; doi:10.1371/journal.pone.0356149)
Supplement: Supplementary file 6 [file pone.0356149.s007.docx]

**Supplementary Table S1. Comparison of the novel single-aortotomy technique and the conventional end-to-side aortic transplantation technique in mice.**

| Technical Feature | New Technique | Conventional Technique |  |  |  |
| --- | --- | --- | --- | --- | --- |
| Recipient aortotomy | Single aortotomy | Two separate aortotomies |  |  |  |
| Number of recipient aortic opening | 1 | 2 |  |  |  |
| Anastomosis configuration | Both graft ends anastomosed to a single recipient aortic opening (end-to-side) | Two independent end-to-side anastomoses |  |  |  |
| Mid-anastomotic site ligation | Yes | Yes |  |  |  |
| Number of transplants | 15 | 15 |  |  |  |
| Donor harvest time (min) | 11.93 [11.4-12.47] | 12.13 [11.44-12.82] |  | p=0.627 |  |
| Recipient preparation time (min) | 10.07 [9.58-10.56] | 10.87 [10.28-11.48] |  | p=0.0331 |  |
| Anastomosis time (min) | 14. 73 [14.09-15.38] | 20.40 [19.59-21.21] |  | p<0.0001 |  |
| Bleeding complications (n) | 0 | 1 |  |  |  |
| Thrombosis (n) | 1 | 1 |  |  |  |
| Graft patency (n) | 14 | 13 |  |  |  |
| Survival rate | 14/15 (93%) | 13/15 (87%) |  |  |  |
| Humane endpoint reached | 0 | 0 |  |  |  |
