# Supplementary material for: A single-aortotomy end-to-side technique simplifies murine aortic transplantation
Source: PLoS One. 2026 Oct 8;21(10):e0356149. doi: 10.1371/journal.pone.0356149 (PMC13649135; doi:10.1371/journal.pone.0356149)
Supplement: Supplementary file 7 [file pone.0356149.s005.docx]

10.6084/m9.figshare.32806397
